# Application of Bioinformatics in Chronobiology Research

**DOI:** 10.1155/2013/153839

**Published:** 2013-09-25

**Authors:** Robson da Silva Lopes, Nathalia Maria Resende, Adenilda Cristina Honorio-França, Eduardo Luzía França

**Affiliations:** Institute of Biological and Health Science, Federal University of Mato Grosso, Rodovia 070, Km 5 s/n 78600-000, Barra do Garças, MT, Brazil

## Abstract

Bioinformatics and other well-established sciences, such as molecular biology, genetics, and biochemistry, provide a scientific approach for the analysis of data generated through “omics” projects that may be used in studies of chronobiology. The results of studies that apply these techniques demonstrate how they significantly aided the understanding of chronobiology. However, bioinformatics tools alone cannot eliminate the need for an understanding of the field of research or the data to be considered, nor can such tools replace analysts and researchers. It is often necessary to conduct an evaluation of the results of a data mining effort to determine the degree of reliability. To this end, familiarity with the field of investigation is necessary. It is evident that the knowledge that has been accumulated through chronobiology and the use of tools derived from bioinformatics has contributed to the recognition and understanding of the patterns and biological rhythms found in living organisms. The current work aims to develop new and important applications in the near future through chronobiology research.

## 1. Introduction 

Living organisms are constantly exposed to cyclical variations that occur throughout the day, night, week, month, and year. These cycles have presented challenges for the survival of all species and have provided mechanisms for development throughout its evolution to anticipate and adjust to the changes that are present in these cycles [1].

As a result of evolution, every living organism carries within itself a clock that regulates its biological functions and determines how it will react to changes and rhythmic cycles, adequately supporting the various functions that develop.

Chronobiology is the science that studies biological rhythms characterized by the recurrent, regular intervals and the cyclic physical, biochemical, and behavioral phenomena that occur in all living organisms [2, 3]. 

There are many types of biological rhythms governing the bodies of living beings, including the frequency domain, which can be classified into ultradian, circadian, and infradian rhythms [4]. 

Circadian rhythms (Latin circa diem, meaning “about a day") have a duration of approximately 24 hours. Rhythms that exceed 24 hours are considered infradian rhythms and include the menstrual cycle and the rate of production of blood platelets. The infradian period can range from approximately seven days to 100 years in the extreme case of the reproductive cycle of Chinese bamboo. Cycles that have duration of less than 24 hours are called ultradian rhythms. These include high-frequency oscillations with periods less than 20 hours, including periods in the order of milliseconds, such as the firing rate of neurons, or in the order of minutes, such as the rhythm of heartbeats [3].

Within the field of chronobiology, there is great interest in understanding how behavioral, psychological, biochemical, and cellular rhythmicity oscillations present within a 24-hour period and how these oscillations are regulated by an internal clock (endogenous).

The circadian rhythms are mainly influenced by endogenous signals, but they adapt to external stimuli (exogenous) from the environment. Temporal integration of the internal and external rhythm is coordinated by the suprachiasmatic nucleus (SCN) of the hypothalamus through the monitoring of temporal signals, called Zeitgebers (a German neologism meaning time marker), which may be temperature, food intake, and the sleep/wake cycle [3, 5].

The circadian rhythm is the most well-studied biological rhythm because it controls the structures responsible for the generation and synchronization of biological rhythms that correspond to 24-hour environmental cycles, that is, day and night. It consists of an oscillatory machine, including a central pacemaker, the SCN in the hypothalamus, and peripheral oscillators located in most tissues and cells, such as cardiomyocytes, fibroblasts, smooth muscle cells, and vascular endothelial cells [6], abdominal adipose tissue [7], and skeletal muscle tissue [8]. 

Briefly, the SCN receives stimuli from the external environment, such as light, which thereafter serves as a synchronizing clock that coordinates internal and external stimuli, such as the light-dark cycle, over 24 hours. The harmony and timing of this system allows the organism to have an anticipatory capacity, enabling it to prepare for events and activities that are basic to sustaining life, such as feeding and resting.

Human circadian rhythmicity is necessary for the functioning of the biological clock, which has central components defined by specific genes, called the “*clock*" genes. The protein products of these genes are essential for the generation and regulation of circadian rhythms within individual cells [9]. The genetic approach has revealed a remarkable conservation of molecular and biochemical features of the biological clock, providing a major breakthrough in identifying the role of each gene.

In mammals, the functioning of the circadian rhythm involves positive and negative feedback mechanisms. The CLOCK and BMAL1 genes (*brain and muscle Arnt-like protein 1*) form a heterodimer that functions as a transcription factor for expression of the gene PER (*period*) and its counterparts 1–3 (Per1, Per2, and Per3), CRY (*cryptochrome*), and the receptor REV-ERB. The PER and CRY genes are mainly transcribed in the morning and are transported from the cytoplasm to the nucleus, where they block the action of CLOCK/BMAL1. When the receptor REV-ERB is absent, the CLOCK and BMAL1 genes are released from this inhibition and are able to begin a new circadian rhythm [10].

Another regulatory mechanism of circadian rhythmicity is the transcription of nuclear receptors REV-ERB (*α* and *β*) and ROR (*α*, *β*, and *γ*), which is activated by the CLOCK/BMAL1 genes. REV-ERB (*α* and *β*) and ROR (*α*, *β*, and *γ*) compete to bind ROREs (*retinoic-acid-related orphan receptor response elements*) present at the promoter of BMAL1. The ROR activates the transcription of the BMAL1 gene, while REV-ERB inhibits the process. Thus, the circadian oscillation of BMAL1 is both positively and negatively regulated [9, 10].

In addition to the environmental Zeitgebers, humans are influenced by social markers. Social commitments, such as work and/or school and social demands, can act as synchronizing agents that function in the establishment of a social rhythm, and these commitments conform to a circadian pattern. Therefore, it can be said that the human biological clock is influenced by three aspects: environmental, social, and biological.

Chronobiology is not specific to humans or animals; it occurs in all living organisms. Studies in plants, such as *Arabidopsis thaliana*, *Oryza sativa* L. ssp. *japonica*, and *Populus trichocarpa*, suggest that a large proportion of the transcriptome is subject to circadian regulation [11]. The idea of the existence for such regulation, which gave rise to the field of chronobiology, had its beginning in approximately 1729, when the astronomer Jean-Jacques D'Ortous of Mairan (1675–1774) showed that the daily rhythm of the opening and closing of leaves in plants was maintained even in constant darkness. After this experiment, others showed that biological rhythms are reflections of environmental fluctuations.

Chronobiology is currently composed of a branch of biomedical sciences that seek to understand the relationships between the rhythms of biological functions, health, and disease. There are other specialties of growing interest within the field of chronobiology, such as chronopharmacology, chronopharmacokinetic, chronoanesthesia, chronoenergy, chronotoxicology, and chronotherapy [1].

It is evident that the circadian rhythm plays an important role in maintaining and coordinating the biological processes necessary for the efficient functioning of the living organism. Modern life often disrupts the workflow of the circadian rhythm in humans. For example, when we travel through different time zones, we suffer from jetlag, also called desynchronization. The symptoms of desynchronization also occur when we have to work on rotating shifts for 24 hours a day. The consequences of frequent jetlag have serious effects on health, including sleep disorders, chronic memory deficits, obesity, diabetes, and other metabolic diseases [7, 12], as well as the development of cancer [13].

Various chronomic studies [6–8, 13–15] seek to understand the endogenous and exogenous mechanisms of synchronization and desynchronization.

However, sleep disturbances and depression symptoms correlated with the time of appearance have been observed since the middle XVIII century. These studies showed that the lack of light affects both mood and physiological functions [3, 16]. These studies were not conclusive because the internal temporal order supplied the elements for the formulation of pathophysiological hypotheses. It was shown that depression and some types of insomnia exhibit changes in human circadian rhythmicity [3, 17].

The posttranslational mechanisms of these genes affect the stability, degradation, and localization of proteins. This can affect metabolic functions related to power and energy metabolism, which may lead to metabolic diseases, such as obesity and diabetes [10].

 Several approaches to biological and genetic models can provide more information about the maintenance of the rhythmicity system and the occurrence of metabolic disorders caused by circadian disorders. The desynchronization of the biological clock resulting from the ingestion of food at an inappropriate time or deprivation of sleep increases the likeliness of metabolic risk and may cause disease [18].

 All these discoveries have contributed to the development of more complex experimental methods, such as the use of “omics" science and the emergence of chronomics.

## 2. Chronomics

The “omics” sciences integrate genomics, transcriptomics, proteomics, and metabolomics to better understand and describe the cellular and molecular mechanisms of living organisms [19]. The mapping of the temporal structure of each variable of biological diversity has led to the development of another “omics” science, chronomics. This designation was first mentioned by Halberg et al. [20], who recommended the use of both experimental procedures and laboratory bioinformatics to map the anticipation of experiences. Chronomics enables quantify the phenomenon as a cycle and its occurrence as a rhythm, making it possible to replicate its events [21].

Since then, many studies of chronomics in medicine have been conducted. Blood pressure, for example, has been prominently featured [21]. Changes in the blood pressure over a complete circadian rhythm can be monitored, and, thus, the chronomics can be used to support the correct diagnosis. This diagnosis can lead to effective treatment and prevent stroke or other diseases [22].

As chronomics has been shown to be effective in diagnosing disease, its use to detect changes that are still reversible may allow for prevention of disease [22, 23].

Bioinformatics and chronomics may provide a tool for mapping the anticipation of the components involved in any biological system or the interaction of these systems. It will be used to improve estimates over a range and to correlate a variable with the multifrequency and the modulations of biological rhythms. Thus, the diagnosis/analysis may be correlated with biological systems with different paces [24].

In chronomics, the main tools used to store, manage, and analyze the events are derived from bioinformatics. Using these tools, chronomes are mapped, grouped, and compared, resulting in the correlation of the multifrequencies and modulations of biological rhythms [25].

Chronomics requires improvements in bioinformatics techniques, including the development of additional tools with features that are adapted to this new area of research.

On the other hand, the variables of biological systems are referred to as the chronome when they are analyzed using the techniques of chronomics. Physiological chronomes and their unique features are calculated within a specific range that is much shorter than the range of the observations of the system as a whole. However, this integrated analysis system is an increment of diagnosis [20, 22–24].

There is evidence that the circadian rhythm involves mechanisms that are independent of the cell nucleus. However, it is also essential to understand the molecular basis for the timing of changes in gene expression through transcriptomics, based on the observation of enzymatic rhythms in the nuclei of red blood cells [26]. More recently, Johnston [27] analyzed the transcriptome of mice for 24 hours and detected the presence of circadian rhythms in adipocytes.

Chronomics, the mapping of chronomes or time structures, aims to provide maps. The research of Löckinger et al. [28] aimed at assessing the circadian time structure of circulating peptides that are secreted by peripheral neurons and have strong effects on blood vessels in 20 healthy, young adults. They concluded that plasma concentrations of vasoactive intestinal peptide and cortisol present circadian rhythms, while substance P and neuropeptide Y undergo variations on the scale of hours.

Another area of “omics” science in which chronomics is particularly important is sportomics [29]. In this area, the focus is on understanding how the rhythms of body temperature are influenced by exercise. There is a proportional correlation between body temperature and the release of the hormone cortisol, a marker of stress [30].

Other studies in this area include investigations of the effects of exercise on sleep patterns, which have been studied since the 1980s. In one of the first epidemiological surveys on sleep and exercise, 1600 respondents aged between 31 and 50 years reported that the quality of sleep and physical performance are impacted by the social and psychological conditions associated with the location used for sleep, the sleep pattern, the lifestyle, and the living conditions of the individual [31].

In a study by Passos et al. [30], the total duration of each stage of sleep and the sleep efficiency were different after acute or chronic physical exercise. It was noted that during the process of adaptation to exercise, the body is restructured and returned to cycling stages of sleep, similar to its state before physical exercise.

In studies on the circadian rhythm response curve comparing exercise and bright light exposure, it was found that physical exercise had a significant influence on the circadian rhythm system, similar to the effects of light [32, 33]. 

All these studies reveal the complexity of the biological functions influenced by endogenous rhythmicity, including the physiological and biochemical mechanisms, as well as exogenous rhythmicity, such as the environment and its changes. Chronomics becomes an area for facilitating the integration of all these variables, and thus organizes the temporal chronomes for better interpretation and understanding.

However, the interrelatedness of the data from both areas is still a challenge for bioinformaticians and chronobiologists. A major barrier to progress is the lack of a proper infrastructure for developing data integration software and research groups.

New tools in bioinformatics have been developed, such as EUCLIS (EUCLOCK Information System), so that new circadian models can be exploited. This system allows the field of chronobiology to exploit the advantages of systems biology research in genetics to investigate the timing of the circadian rhythm at the level of the genome, the transcriptome, the proteome, and the metabolome [15]. 

EUCLIS takes advantage of the advanced architecture of the database that is used in pioneering liver cell research, the HepatoSys. According to Roennberg [13], this framework divides the database into several distinct modules: the experiments base contains the experimental procedures and provides a visualization tool for time series data; the knowledge base is a digital library that contains modules for common components used by the experiments base; the models base categorizes experimental models in humans, rodents, and birds; the genes base provides a catalog of genes associated with circadian rhythms in experimental models, including homologous sequences, the functions of genes, genes associated with metabolic pathways, and their phenotypes, chromosomal locations, and the correlated literature; the references base is a repository of references including lessons and materials for chronobiological study; the tools base contains software used for simulation and analysis of experimental data to produce graphs and images; and the image base provides a repertoire of images and their metadata.

EUCLIS also provides a place to document events within the community through the museum base, as well as a place to document scientific genealogy through the pedigree base [13, 15].

EUCLIS was developed by chronobiologists in the European community, known as EUCLOCK [14], and was created to facilitate interaction among researchers. This community originated in January 2006 through the integration of the efforts of 34 chronobiology laboratories at 29 institutions in 11 European countries over 5 years. This network integration helped provide an understanding of how circadian clocks are synchronized with the rhythm of a particular environment, whether endogenous or exogenous. For this purpose, the researchers used the most advanced methods of phenotypic and functional genomics and developed innovative techniques to measure the periodicity of the circadian CLOCK genes in human skin fibroblasts and the phase of the circadian CLOCK genes in the oral mucosa and blood leukocytes, thus aiming at the development of diagnostic tools for diseases related to circadian rhythms [13].

The contributions of information technologies are indisputable, especially biotechnology and bioinformatics, for the treatment, storage, management, analysis, and visualization of large amounts of data compiled from the databases within the “omics" sciences.

## 3. Artificial Intelligence Techniques

Bioinformatics is an interdisciplinary field of study that has recently emerged and that involves physical and chemical biology, cellular and molecular biology, mathematics and computer science. The main goal of bioinformatics is to resolve problems arising from bioscience using computer science techniques, such as artificial intelligence, data mining, neural networks and Bayesians, and evolutionary algorithms.

Studies on physical performance also employ bioinformatics. With this science, it is possible to understand how each pathway of a particular process is working under conditions at rest or in different situations, such as during physical stress or even a pathological state. Thus, each compound and its related interactions can be represented on a metabolic map (integrated representation of human metabolism), providing a complete map of the metabolome, which we call a sportomic. The metabolic responses to exercise are dynamic and influenced by time. Due to the large amount of data derived from this study, the tools of bioinformatics, MarkerLynx (Waters Corporation, USA) and ChromaLynx (Waters Corporation, USA), were used for identification and quantification of the relevant compounds for the metabolomic analysis in accordance with a database from the National Institute of Standards of Technology (NIST) library [29, 34].

The modern technologies used for high-throughput measurements in molecular biology, such as microarray and RT-PCR, produce a large amount of data. With these large datasets, the emphasis has been to move from traditional statistical tests to new methods of data mining [35], which derive their roots from statistics, artificial intelligence, and machine learning [36].

## 4. Data Mining

Data mining focuses on automating the discovery of knowledge that is contained within a database but that is not readily perceptible. The use of data mining in a database is considered the central step in a larger process called knowledge discovery in databases (KDD), which includes several other processes that can be divided into preprocessing and postprocessing steps [37].

KDD preprocessing includes several steps, such as integration, cleansing, data discretization, and the selection of relevant attributes for the task of data mining. Postprocessing through KDD improves the understanding of the results obtained from data mining.

There are different data mining tasks, each of which aims to obtain a certain type of knowledge that is associated with a specific problem. The main tasks performed by DM algorithms are the development of clustering, association, and dependency models. These tasks can discover hidden associations or sequences within datasets, as well as clustering and visualizing the relationships among these data, enabling the prediction of hidden patterns.

Data mining can also be used in studies of gene expression rhythms [35]. Chronobiological studies used data mining or, more specifically, the task of clustering, to extract patterns from data to related behaviors and gene expression patterns in humans, rodents, and plants [7, 8, 11, 12].

Clustering involves dividing a dataset into groups based on the measurement of one or more characteristics of the data. Thus, it combines the elements with the same characteristics into different groups [38].

Filichkin et al. [11] showed that investigation of the cyclical transcriptome of plants could help elucidate the similarities and differences, as well as the common points of regulation in monocotyledonous and dicotyledonous plants. For this purpose, the authors used a combination of oligonucleotide microarrays and data mining to examine the daily rhythms in the gene expression of rice (*Oryza sativa* ssp. *japonica*) and poplar (*Populus trichocarpa*). The transcriptomic data related to rhythm were observed in different periods and under varying temperature conditions.

In the same study, the results identified groups of coexpressed genes at specific times of the day and showed that the rhythmic expression of the transcriptome of rice and poplar spanned all hours of the day, with peak expression levels at dawn and dusk [11].

Other studies have described the regulation of circadian gene expression in skeletal muscle [8], white adipose tissue, [7] and lungs tissue [12] of the rat. 

In a study by Almon et al. [8], mRNA expression was observed in the gastrocnemius muscles in a series of 54 animals that were euthanized at different times within the 18 cycle of 24 hours. Mining of such data identified 109 genes that were expressed with rhythmicity and grouped them into eight distinct categories corresponding to 11 functions in the context of temporal expression.

In the study by Sukumaran et al. [7], circadian oscillations in gene expression in the white adipose tissue of mice were identified, and the regulatory role of circadian timing in the function of this tissue was examined. In total, 190 sets of probes that showed different circadian oscillations were identified through mining of the microarray data. These circadian probes sets were divided into seven different temporal groups in which >70% of the genes showed maximum expression during wakefulness (dark). These genes were grouped into eight functional categories that were examined in the context of their temporal expression. The circadian oscillations were also observed in the measurement of plasma leptin, glucose, insulin, corticosterone, triglycerides, free fatty acids, and LDL cholesterol. This oscillation of physiological circadian rhythms together with the functional classification of these genes suggests an important role for circadian rhythms in controlling various functions in white adipose tissue, including adipogenesis, energy metabolism, and the regulation of the immune system.

Similar work was conducted by Sukumaran et al. [12] to determine the patterns of gene expression in rat lungs during the light-dark cycle. In this work, 646 genes that showed variations in expression were identified through data mining, and eight different temporal groups were analyzed. More than two-thirds of the probes showed peak expression during the cyclic period of light or dark. Many of the genes whose expression peaked during the light were related to the extracellular matrix, the cytoskeleton, and protein processing, which seem to be mainly involved in tissue repair and remodeling.

Several studies have been conducted using data mining tools in the area of health for understanding pathological mechanisms. A study by Wood et al. [38] used data from Oncomine, an online microarray database that contains a set of gene expression data from various human cancers along with normal tissue controls. These data were analyzed to compare the levels of PERIOD gene expression (Per1, Per2, and Per3) in colon cancer and rectal and intestinal adenomas. The results showed that the role of these genes in circadian desynchronization can be used in cancer therapy based on nocturnal exposure to light.

## 5. Artificial Neural Networks and Rule-Based Systems

Beyond data mining, other artificial intelligence techniques, such as artificial neural networks [39, 40] and rule-based systems [41, 42], have contributed to the study of chronobiology. 

An artificial neural network is a parallel processing system that has nonalgorithmic computation as its main feature, which is reminiscent of the structure of the workings of the human brain neurons [43].

In troubleshooting this technique, the model initially passes through a learning phase in which a reduced set of examples is presented to the network. Then, the network automatically learns the characteristics required to represent the information provided and therefore recognizes any existing standard that can be used to later identify consistent data in an unknown data set.

In recent years, Artificial Neural Networks have received considerable attention as a computing tool in several areas, including the learning decision process, prediction problems, pattern similarity discovery, data filtering, automatic acquisition of knowledge, monitoring and rapid diagnosis, and incomplete information processing, among others.

In chronobiological studies, artificial neural networks were used to identify patterns in the circadian rhythms of humans. The work of Kolodyazhniy et al. [40] presented an approach to a regression nonlinear model based on artificial neural networks with the aim of recognizing patterns in the circadian rhythms of 25 healthy young humans. The data were derived using an ambulatory multichannel monitoring system during daily routines for one week. The devices collected large amounts of data related to physiological, behavioral, and environmental factors, including body temperature, cardiovascular and respiratory functions, motion, posture, local temperature, light intensity perceived at eye level, and sleep [44].

 The results showed that this type of neural network significantly enhances the variance of error prediction compared with traditional approaches for determining the circadian phase based on individual indicators (body temperature, acceleration of movement, and sleep recording). Two sets of noninvasive measures were identified that, combined with the predictive model, can provide researchers and clinicians with an accurate measure of endogenous time. This method was validated in healthy young men and requires testing in a clinical population or people who suffer from sleep/wake disturbances.

In studies conducted by Jara et al. [42], an intelligent system to detect and predict myocardial diseases by analyzing the electrocardiogram curve was developed. 

Intelligent systems seek to simulate the knowledge of an expert in the subject through a rule base that is used to review the information presented to the system. The system developed by Jara et al. [42] first detects the symptoms and then makes the prediction of the disease. The system presents an algorithm that is based on chronobiological studies demonstrating that a myocardial infarction could be predicted up to eight days before it occurs.

This study is based on the assumption that the rate of the heartbeat of a patient is highly variable, and it was necessary to perform monitoring for 24 hours. For each hour, the maximum and minimal heart rates were recorded. With these data, the system's intelligence-based Jess rules determined the difference between the minimum and maximum heart rate, which indicated a risk for myocardial disease. The authors emphasize the importance of further tests before substantiating a positive diagnosis.

**Figure 1:**
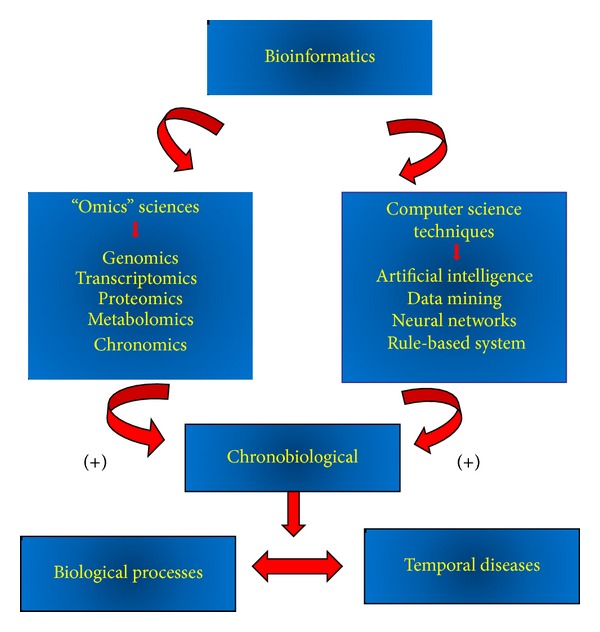
Relationship between bioinformatics, chronobiological, and diseases.

Based on these studies, it is clear that the various techniques originating from computer science and integrated with the knowledge of cell and molecular biology, physiology, chemistry, medicine, and bioinformatics have assisted in the study and comprehension of how biological rhythms of living organisms influence the neuro-immuno-endocrine response. The results have become more consistent and indispensable as the amount of data generated in studies of the “omics" sciences and computer science techniques has increased. The variables involved are complex and numerous and it is possible that the bioinformatics and chronobiological can be applicable in understanding the biological rhythms and various diseases (Figure 1). 

The observation and recording of endogenous and exogenous biological rhythmicity is the focus of chronomics in collaboration with bioinformatics. This approach will contribute to improvements in the diagnosis of disease and understanding the pathophysiology and treatment of diseases, as well as the temporal variables of disease, based on quantitative and qualitative interpretations of various disorders. The fields of chronomics with bioinformatics are applicable not only in diagnosis but also in understanding the chronomes according to an individual's rhythm.

## 6. Conclusion

In recent decades, chronobiology has revealed the existence of a temporal regulation system that synchronizes all body systems to environmental cycles, such as the day-night cycle. Therefore, it is now known that living organisms respond proactively to environmental rhythmicity and therefore actively prepare.

Although considerable progress has been achieved in the understanding of the biological rhythms in the SCN and its synchronization with the peripheral tissues and exogenous signals, many questions remain about the functioning and dynamics of biological rhythms and how they influence and are influenced by genetics and phenotype.

The studies of the “omics" sciences produce a large amount of information on the molecular biology of various living organisms, which is used for the production of new scientific knowledge through different experimental paradigms. The in silico approach is represented by methodologies based on the creation of algorithms and bioinformatics tools.

Maps of chronomes in circadian and noncircadian patterns may be useful for screening, diagnosis, and a better understanding of the cellular and molecular mechanisms of life. The complex maps of chronomes should be made available to the scientific community online so that investigators may study the effects of gender, age, ethnicity, patterns of behavior, and geographic location as a function of time.

Bioinformatics, as well as other well-established sciences, provides an approach based on scientific methods for the analysis of data generated by “omics" projects that can be used in studies of chronobiology.

Some of the studies presented in this paper made use of various bioinformatic techniques, such as data mining, artificial neural networks, and rule-based systems, to extract patterns in the rhythms of CLOCK gene expression and understand how these rhythms can influence rhythmic oscillations in the peripheral tissues. The results of studies that apply such techniques show how they have significantly aided the understanding of chronobiology.

However, no bioinformatics tool is sufficient to eliminate the need for an understanding of the field of research or the data to be manipulated, nor can such tools replace analysts and researchers. For the application of bioinformatics tools, such as the KDD, in which preprocessing is necessary to select the relevant data from a repository and perform cleanup and discretization, the intrinsic knowledge of researchers and scientists in the field is extremely important. Similarly, in postprocessing KDD, it is often necessary to conduct an evaluation of the results of mining to determine the degree of reliability.

It is evident that our knowledge has already increased in the field of chronobiology, and the use of tools derived from bioinformatics can contribute to the recognition and understanding of the patterns in the biological rhythms of living organisms. New and important applications for chronobiology will be developed in the near future.
